# Advances in Organosulfur-Based Polymers for Drug Delivery Systems

**DOI:** 10.3390/polym16091207

**Published:** 2024-04-25

**Authors:** Fawad Islam, Qingle Zeng

**Affiliations:** College of Materials, Chemistry & Chemical Engineering, Chengdu University of Technology, Chengdu 610059, China

**Keywords:** organosulfur polymers, drug delivery, polymerization, nanotechnology, therapy

## Abstract

Organosulfur-based polymers have unique properties that make them useful for targeted and managed drug delivery, which can improve therapy while reducing side effects. This work aims to provide a brief review of the synthesis strategies, characterization techniques, and packages of organosulfur-based polymers in drug delivery. More importantly, this work discusses the characterization, biocompatibility, controlled release, nanotechnology, and targeted therapeutic aspects of these important structural units. This review provides not only a good comprehension of organosulfur-based polymers but also an insightful discussion of potential future prospectives in research. The discovery of novel organosulfur polymers and innovations is highly expected to be stimulated in order to synthesize polymer prototypes with increased functional accuracy, efficiency, and low cost for many industrial applications.

## 1. Introduction

The field of drug delivery systems is one of the most important ways to make drugs more effective as medicines. With the huge number of compounds available, organosulfur-based polymers have become highly desired. They have become attractive to researchers because they are naturally biocompatible, flexible, and easy to customize. This review aims to explore how modern researchers are using organosulfur polymers for developing drug transport frameworks. Currently, there is a lot of interest in the pharmaceutical sciences because researchers ought to find new routes for drug delivery. Due to their unique and excellent properties, organosulfur-based polymers have become promising entities for designing these structural scaffolds. This work thoroughly examines the progress made and plausible futuristic research directions in this area. Polymers based on organosulfur moieties have a lot of different qualities that make them useful for medicine delivery packages [1]. Their natural biocompatibility means that there will be no undesired side effects when working with plant systems, which makes them suitable for specific medical uses. These polymers also have the potential to alter their physical properties, thus making it possible to create custom drug delivery systems for specific therapeutic needs. These properties, along with the fact that these are biodegradable, not dangerous, and do not permit controlled drugs release, make organosulfur-based polymers excellent entities in the field of drug delivery.

One of the most beneficial aspects of polymers based on organosulfur is their biocompatibility within biological systems. Organosulfur-based polymers have shown a high level of biocompatibility, with minimal inflammatory response, as demonstrated by in vitro studies with human macrophages [2]. This quality has been well established after long-term implantation in rabbits for sustained insulin delivery [3]. The fact that these polymers break down naturally also adds to their safety since they often metabolized into non-harmful units, such as urea, which ultimately restrict them from making other higher biomolecules. Drug delivery systems need to be safe for a long time, and the use of organosulfur polymers eliminates the long-term accumulation risk associated with non-degradable polymers, as observed in studies comparing polystyrene and poly(L-cysteine) implants [4].

Polymers based on organosulfur offer a wide range of possibilities for drug delivery due to their exceptional flexibility. Medical professionals can tailor these polymers to fit specific types of medicines, delivery methods, and delivery strengths. Medical professionals can tailor organosulfur-based polymers to meet their exact needs, whether this entails delivering hydrophilic or hydrophobic medicines, small particles, or biologics [5]. These polymers have diverse applications beyond similar remedies. Changing the molecular weight, branching, and bonding of the polymer can alter the rate at which the drug is released. Such variations are necessary for better pharmacokinetic studies, which make sure that the right amount of medicine stays in the body for the right amount of time.

Controlled drug release is an important part of medical drug delivery systems because it directly affects the safety and effectiveness of the medicine. Compared to traditional polymers, organosulfur-based polymers can be synthesized with diverse functional groups allowing for precise control over drug release kinetics and responsiveness to specific stimuli. Controlling the release rate of drugs is particularly beneficial for drugs that require continuous release to maintain high healing levels within a short healing window. Thioether-containing polymers like polypropylene sulfide exhibit pH-responsive release, ideal for delivering drugs to the acidic environment of the stomach. The addition of other functionalities that respond to specific stimuli can change the release pattern, i.e., zero-order, first-order, or pulsatile release. Pharmacists can modify the drug delivery to according to the type of target, illness, specific groups of people, etc., to meet research requirements [6]. Conducting thorough preclinical and medical tests to check the safety and effectiveness of organosulfur polymer-based drug delivery systems is an important step in finding their efficacy in clinical settings. The designing of drug transport structures using organosulfur polymers is an exciting area since these are biocompatible, flexible, and adaptable and can be used in sustainable drug release to thus improve the potency of drugs. As research in this area continues to flourish, it is believed that new drug transport methods that use the unique properties of organosulfur-based polymers will have a big impact on the pharmaceutical industry and be beneficial to patients all over the world. 

## 2. General Synthetic Techniques

### 2.1. Ring-Opening Polymerization

**Scheme 1:**
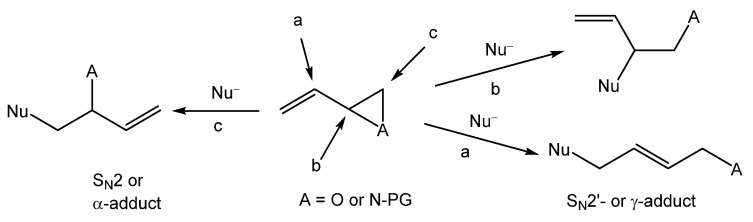
Ring-opening reactions of small-ring heterocycles.

By breaking open cyclic sulfides (thiiranes) with diverse nucleophiles like amines or thiols, this polymerization method improves the controlled growth of polymer chains [7]. The reaction in Scheme 1 is a general representation of an epoxide ring-opening reaction, aziridine or oxetane ring-opening, among many others linked to ring-opening polymerization. The nucleophile could be number of different chemicals, such as amines or thiols. This method is efficient for the synthesis of polymers with fixed molecular weights and low polydispersity, and it yields reproducible high-quality output. Specific cyclic sulfur-based monomers along with initiators or catalysts are important in executing and speeding up the polymerization process.

### 2.2. Thiol-Ene Click Chemistry

**Scheme 2:**
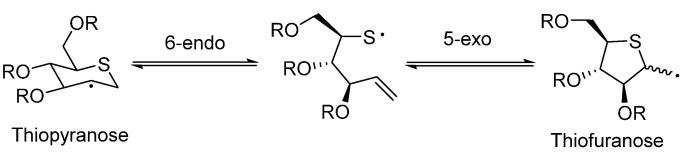
Thiol-ene click reaction.

For the objective of synthesizing polymers, this method involves the interaction between a thiol functional group and an alkene (Scheme 2) [8]. The synthesis of polymers based on organosulfur can be accomplished using this method, which is both extremely effective and flexible. In addition to having a rapid reaction rate and excellent yield, it provides a large amount of efficiency and selectivity. This enables the synthesis of polymers that involve different structural and functional patterns since it does not require drastic reaction conditions and is suitable for a wide variety of various substrates.

In addition to limiting the selection of monomers, the requirement of thiol- and alkene are important. If the reactions are not well regulated, they have the potential to cause side reactions or cross-linking.

### 2.3. Radical Polymerization

**Scheme 3:**
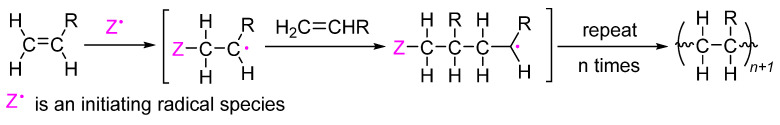
Example of radical polymerization.

This method accomplishes the initiation of the polymerization reaction through the use of radical initiators (Scheme 3). This approach can be used to synthesize polymers from monomers with sulfur-containing functional groups [9], hence offering an easy synthetic procedure. This mechanism enables the precise manipulation of the structure and synthesis of polymers with high molecular weights. However, the small-scale reactions can lead to a more extensive distribution of molecular weights and less control over the dispersity of the polymer. It is possible that the utilization of radical initiators, which may result in unfavorable reactions, is required, and the susceptibility to oxygen and other contaminants can lead to unwanted polymerization.

### 2.4. Polycondensation

**Scheme 4:**
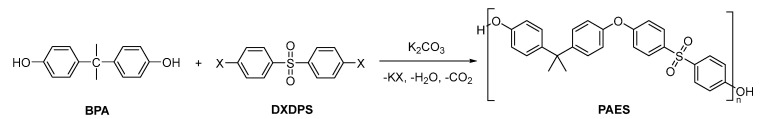
Polycondensation reaction.

This method involves the condensation reaction of one or more specific monomers with the removal of a minor molecule, such as water or alcohol [10]. Through the use of monomers that contain sulfur-containing specific compounds, it is possible to synthesize organosulfur-based complete polymers using polycondensation reactions (Scheme 4) [11].

In addition, this type of synthesis facilitates the production of polymers with high molecular weights and precise thermal equilibrium. It offers flexibility in the selection of monomers and valuable structures and is applicable in the production of both straight and branched polymers. To prevent side reactions or unintended chain termination, it is necessary to carefully control reaction conditions such as temperature and pressure. The synthesis and purification of the polymers can be hindered by the development of byproducts during the reaction [12]. The presence of barriers related to the availability and reactivity of monomers is possible.

### 2.5. Emulsion Polymerization

Mixing monomers with reacting groups that contain sulfur with surfactants and other ingredients in a water-based medium is part of this method. Polymerization then forms latex particles, which can also be converted into polymers based on organosulfur [13]. It is possible to make pure polymers with a narrow particle length distribution using an emulsion-based approach. This type of reaction provides good control over the shape and location of particles on surfaces and is beneficial in mass production.

R_p_ = K_p_[M] (N_micelles_/2N_A_) (1)

whereby R_p_ is a rate of reaction and K_p_ is the constant.

The use of specific surfactants and stabilizers is necessary, which may need to be removed after the reaction is completed. Reaction conditions such as temperature and pH can affect this reaction by potentially altering the size and balance of the particles. Latex particles can only be produced using this method, and they may require similar processing to obtain the desired organosulfur-based total polymers [14].

## 3. Specific Synthetic Techniques

### 3.1. Click Chemistry

**Scheme 5:**
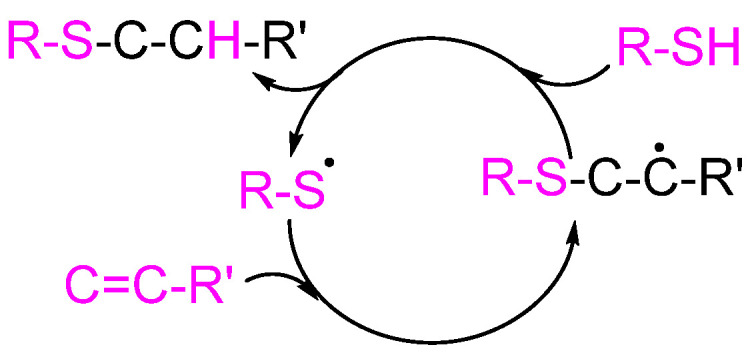
Thiol-ene click polymerization.

Click chemistry has become a powerful way to make different kinds of polymers, like total polymers based on organosulfur. Click chemistry processes, such as thiol-ene click chemistry and thiol-one click chemistry, are environmentally friendly and selective ways to make polymers. These processes are useful in producing a higher yield and good control over the structure of sulfur-containing polymers (Scheme 5) [15] due to the quick and selective nature of click reactions. These polymers find applications in the synthesis of drug delivery structures, tissue engineering scaffolds, and coats due to their controllable structure and non-toxic nature.

### 3.2. Controlled or Living Polymerization

**Scheme 6:**
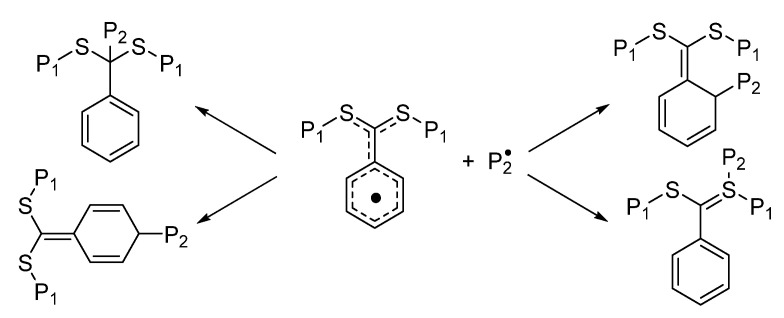
An example of controlled/living polymerization.

The approaches of reversible addition−fragmentation chain transfer (RAFT) polymerization and atom transfer radical polymerization (ATRP) have been expanded to include the synthesis of polymers largely based on organosulfur. These methods allow precise control over the polymerization process that make polymers with clear structures, controlled molecular weights, and limited polydispersity (Scheme 6) [16]. The use of sulfur-containing monomers, like vinyl monomers, in controlled polymerization methods can make organosulfur-based polymers with specific properties. These polymers find applications in controlled drug delivery systems, sensors, and membranes, where controlling the polymer structure is essential for achieving desired characteristics [17]. The precise manipulation of polymer structures is crucial for achieving desired characteristics in applications such as controlled drug delivery systems, sensors, and membranes.

### 3.3. Electrochemical Polymerization

**Scheme 7:**
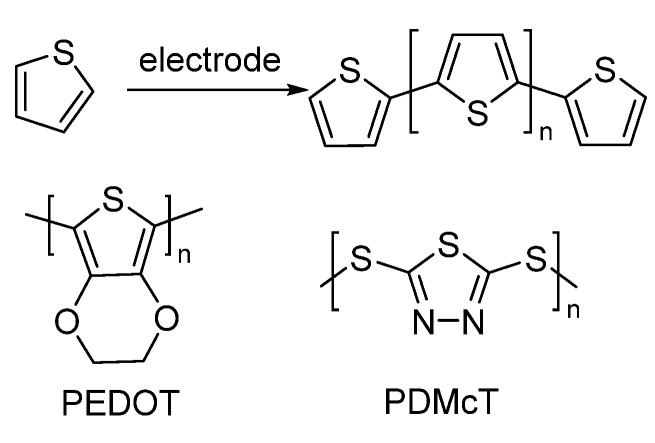
Example of electrochemical polymerization and some typical sulfur-containing polymers.

Another new method of synthesizing polymers is through electrochemical polymerization, which uses electrochemical processes [18]. This method has benefits like mild reaction conditions, control over polymer structure, and the ability to include monomers that contain sulfur. Scientists have used electrochemical methods to prepare organosulfur-based polymers for drug delivery systems [19,20]. Organosulfur-based polymers that are electrochemically mixed can also be used in energy storage devices like batteries and supercapacitors (Scheme 7). During the controlled electrochemical polymerization process, the process puts together polymers with advanced conductivity, high stability, and better electrochemical qualities.

### 3.4. Supramolecular Assembly

In supramolecular assembly methods, small molecules or polymers react with each other on their own through non-covalent interactions, such as hydrogen bonding or π-π stacking [21]. Supramolecular meetings enable the synthesis of organosulfur-based polymers with specific structures and functions. This can be achieved by incorporating sulfur-containing components into the molecular structure. With this method, many unique properties of the polymers can be created and controlled in various ways. These polymers find uses in sensors, optoelectronics, and catalysis, where the self-assembly process can create new systems of improved performance [22].

### 3.5. Hybrid Polymerization Strategies

**Scheme 8:**
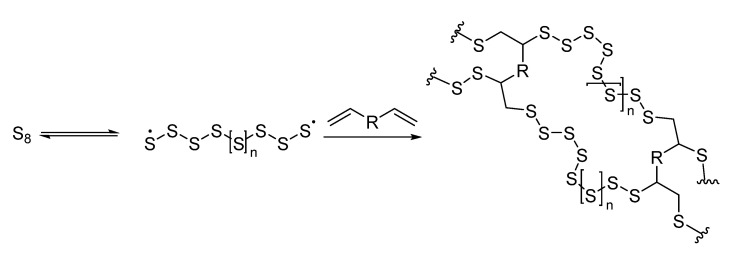
Hybrid polymerization.

Hybrid polymerization uses a combination of different polymerization methods to make complex polymers; e.g., ring-starting and controlled/living polymerization methods can be used together to generate desired organosulfur-based total polymers with controlled molecular weights and structures (Scheme 8) [23]. By using more than one polymerization method, researchers can synthesize a wide range of the structures and features of organosulfur-based polymers. Scientists can use the different polymerization methods to incorporate various functions and customized features into these polymers, enabling their application in drug delivery systems, gene delivery, biomaterials, and nanotechnology [24]. This method is useful in many areas, such as biomedical, electronics, and materials technology [25,26].

## 4. Specific Drugs Delivered Using Organosulfur-Based Polymers

**Doxorubicin** is an extensively used chemotherapy drug for the treatment of numerous cancers, including breast cancer, lung cancer, and lymphomas. These polymers act like Trojan horses, encapsulating doxorubicin while a high concentration of glutathione (GSH) acts as a key inside tumors. It cleaves the disulfide bonds, releasing the drug directly into the cancer cell, maximizing its impact while minimizing harmful side effects. This thiol-cleavage mechanism is a precise chemical maneuver orchestrated by the tumor’s own environment. The use of organosulfur-based polymers can help deliver doxorubicin to the right place, improving its balance, solubility, controlled release, therapeutic effectiveness, and side effects [27].

**Scheme 9:**
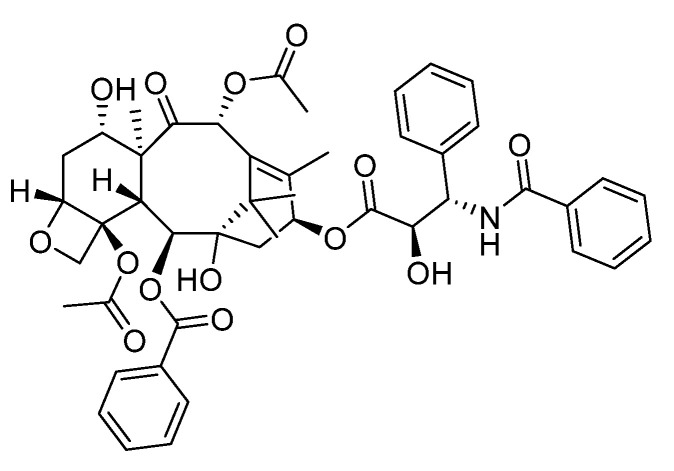
Paclitaxel.

**Paclitaxel** is a robust anti-cancer drug used to treat ovarian, breast, and lung cancers (Scheme 9) [27]. However, it has lower solubility and might cause severe side effects. These polymers molecules confine paclitaxel due to hydrophobicity along with hydrophilic water to create a cozy micelle-like environment, dissolving paclitaxel and enhancing its delivery. Additionally, they act like timed-release capsules, gradually releasing the drug for sustained effectiveness, reducing the need for frequent doses and minimizing side effects. By formulating paclitaxel with primarily organosulfur-based polymers, its solubility may be advanced, and a sustained release can be carried out, leading to more advantageous therapeutic effects and decreased toxicity.

**Insulin** is a hormone used to treat diabetes. The transport of insulin through subcutaneous injections may be challenging because of its instability and quick half-life. Primarily organosulfur-based polymers may be designed to encapsulate and protect insulin, considering sustained release and improved stability, enabling extra powerful and convenient insulin delivery [28,29]. Poly(ethylene glycol)-b-poly(methacryloyl-cysteine) polymers were synthesized and loaded with insulin. The polymers were capable of protecting insulin from enzymatic degradation in the stomach and releasing it in a controlled manner in the presence of glutathione in the small intestine [30].

**Scheme 10:**
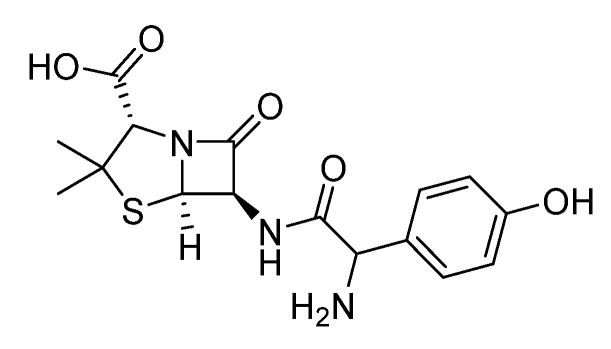
Antibiotic: amoxicillin.

**Antibiotics:** Harmful bacterial infections are often treated with antibiotics such as gentamicin, amoxicillin (Scheme 10), and ciprofloxacin [31]. However, antibiotic resistance development and low bioavailability can compromise their effectiveness. Organosulfur polymers step in like armor, encapsulating antibiotics and protecting them from enzymes that would render them inactive. Additionally, they can be designed to target specific bacterial biofilms, the sticky havens where resistant bacteria thrive. By concentrating the attack on these biofilms, organosulfur polymers empower antibiotics to overcome resistance. Antibiotics can be encapsulated in organosulfur-based polymers, which improve their solubility, stability, and targeted delivery, making them more effective as medicines.

**Anti-inflammatory drugs:** The long-term use of non-steroidal anti-inflammatory drugs (NSAIDs) can cause gastrointestinal consequences. Organosulfur polymers play the peacemaker. They can be linked to NSAIDs and applied with pH-responsive properties. These polymers remain inactive in the acidic stomach but readily release the drug once they reach the more neutral environment of the intestine. By formulating those pills with organosulfur-based polymers, a sustainable release can be achieved, reducing dosing frequency and minimizing gastrointestinal infection.

## 5. Characterization of Organosulfur Polymers

Characterization is critical to understand the characteristics and overall performance of organosulfur-based polymers for drug delivery systems. By using diverse analytical techniques, researchers can gain insights into these polymers’ structural, morphological, thermal, and mechanical properties. This segment introduces a few generally used techniques for characterizing organosulfur polymers and discusses their benefits and limitations [32].

One of the essential techniques used for characterizing organosulfur polymers is Fourier-transform infrared spectroscopy (FTIR). FTIR offers facts about the functional nature of the polymer structure by measuring the absorption of infrared radiation. This technique allows researchers to observe unique chemical bonds and verify the successful synthesis of organosulfur polymers. Additionally, FTIR can provide insights into the degree of linking or branching in the polymer network [33].

Nuclear magnetic resonance (NMR) spectroscopy is another commonly used method. NMR spectroscopy allows the evaluation of the polymer’s molecular structure, presenting information about the polymer chains’ connectivity, stereochemistry, and composition. Combining proton NMR (^1^H NMR) and carbon-13 NMR (^13^C NMR) techniques helps researchers fully understand the chemical shape and arrangement of the organosulfur polymer.

Differential scanning calorimetry (DSC) and thermogravimetric analysis (TGA) are two important thermal analysis methods for studying the thermal properties of organosulfur polymers. DSC measures the warmth flow related to segment transitions, melting factors, glass transition temperatures, and the thermal balance of the polymers. TGA then measures the weight loss as a characteristic of temperature, offering records of the polymer’s thermal degradation conducts and thermal stability [34].

Researchers can study the morphology and base characteristics of organosulfur polymers using scanning electron microscopy (SEM) and atomic force microscopy (AFM). SEM gives high-resolution images of the polymer surfaces, allowing researchers to observe the morphology, particle size, and distribution. AFM offers three-dimensional images of the polymer surface, permitting the analysis of surface roughness, topography, and nanoscale functions.

Techniques like tensile testing or dynamic mechanical analysis (DMA) can determine mechanical properties such as tensile strength, elasticity, and flexibility. Tensile testing measures the mechanical reaction of the polymer under applied stress, presenting facts about its energy and deformation behavior. DMA tests the viscoelastic properties of the polymer by putting it under oscillating stress or strain [35].

While these strategies are precious for characterizing organosulfur polymers, it is essential to recognize their barriers. For instance, FTIR and NMR require natural samples and may not describe the polymer’s spatial distribution or heterogeneity. Surface evaluation is limited to SEM and AFM, and artifacts may be introduced by the pattern practice method. Additionally, the mechanical properties determined through tensile testing or DMA might only partially represent the conductivity of the polymer in complicated physiological environments [36].

X-ray diffraction (XRD) is used to research a material’s crystalline structure, including organosulfur polymers. By exposing the polymer to X-ray radiation, researchers can obtain diffraction patterns that provide data about the association and orientation of polymer chains. XRD can assist in determining the degree of crystallinity, crystal size, and the presence of any crystalline levels inside the polymer.

Researchers use gel permeation chromatography (GPC) or size exclusion chromatography (SEC) to determine the molecular weight distribution and average molecular weight of polymers. Using a porous column to separate the polymer chains by length, researchers can look at the elution pattern and figure out parameters like the polydispersity index [37], the weight average molecular weight, and the quantity common molecular weight [38]. GPC/SEC gives insights into the polymer’s molecular length and distribution.

Dynamic light scattering (DLS) is a technique used to analyze the dimensions and length distribution of particles or macromolecules. By measuring the depth fluctuations of scattered light due to Brownian motion, researchers can determine the hydrodynamic diameter of organosulfur polymers [39]. DLS is mainly beneficial for analyzing the size and stability of polymer nanoparticles, or micelles, in drug delivery systems.

Differential Scanning Calorimetry (DSC) offers records of the approximate thermal transitions, along with the glass transition temperature, melting factor, and crystallization conduct of organosulfur polymers. DSC can also study the compatibility and phase separation of polymer blends or the thermal behavior of drug-loaded polymer systems [40].

EPR spectroscopy is a way to examine the electronic structure and residence of substances, such as organosulfur polymers. By finding and studying the absorption and emission of microwave electromagnetic radiation, EPR spectroscopy can help us understand the presence of radical species, paramagnetic centers, and unpaired electrons in the shape of the polymer.

## 6. Properties of Organosulfur-Based Polymers

Organosulfur polymers show inherent biodegradability, making them suitable for drug delivery programs. The sulfur in the polymer spine creates angled links that can break down in certain physiological conditions or when certain enzymes are active. This feature ensures that the polymers may be damaged into biocompatible byproducts, lowering the danger of long-term accumulation and potential toxicity [41]. Organosulfur polymers are biodegradable, allowing for the tailored control of medication release by adjusting the degradation rate to match the desired drug release profile.

For instance, in the case of disulfide bonds, the presence of a decreasing environment, including intracellular situations, can result in the cleavage of these bonds, resulting in the release of the encapsulated drug. This ability to respond to particular stimuli allows unique management over drug release, improving healing efficacy and minimizing side effects.

Moreover, organosulfur polymers can potentially encapsulate many drugs, including hydrophobic and hydrophilic compounds especially with thiol (-SH) or sulfide (-S-) which are interacted strongly through covalent or non-covalent interactions [42]. These interactions permit efficient drug loading and encapsulation in the polymer matrix. The high medication stacking capacity and embodiment productivity of organosulfur polymers make them efficient and targeted delivery systems.

Products containing sulfur can affect the drug release kinetics of organosulfur polymers. The presence of sulfur can affect the hydrophobicity or hydrophilicity of the polymer, modifying the therapeutic potential. For instance, adding hydrophobic sulfur-containing groups can create a hydrophobic center inside the polymer, which is essential for the long-term release of hydrophobic drugs. Alternatively, introducing hydrophilic sulfur-containing parts can improve the water uptake and swelling behavior of the polymer, facilitating the release of hydrophilic drugs [43]. Researchers can change the drug release kinetics of organosulfur polymers to obtain the desired healing effects by changing the type and amount of sulfur-containing functional groups.

### 6.1. Stability in Physiological Conditions

An Assessment of the Physiological Parameters: Stability Concerns and Strategies for Organosulfur Polymers in Cancer and Diabetes.

The allure of organosulfur-based polymers for biomedical applications stems from their unique blend of properties, including tunable biocompatibility, controlled degradation, and diverse functionalities. However, diseases such as cancer and diabetes present formidable challenges to their stability. This intricate interplay between polymer chemistry and the physiological environment demands careful consideration.

Oxidative cleavage and enzymatic challenges: Both cancer and diabetes are characterized by elevated levels of reactive oxygen species (ROS), acting as relentless oxidants ready to cleave sulfur-containing bonds within the polymer backbone [44,45]. Additionally, proteases and lipases residing in biological fluids pose a constant threat, eager to cleave ester or amide linkages, potentially dissecting the polymer structure [46]. Even the seemingly harmless aqueous environment can prove deceitful as hydrolysis can quietly attack certain linkages, compromising the polymer’s integrity [47]. To cope with such challenging conditions, researchers have devised inventive strategies. Fortifying the polymer design with robust sulfur-containing monomers and incorporating cross-linking tactics boost the backbone’s defenses against oxidation [40]. Shielding the vulnerable exterior with hydrophilic or zwitterionic moieties acts as a diplomatic shield, appeasing the enzymatic effects and enhancing biocompatibility [48]. Encapsulating therapeutic agents within the polymer matrix, akin to cloaking them in a protective fortress, shields them from degradation while enabling controlled release at the target tissue, maximizing their therapeutic impact [49].

### 6.2. Toxicity of the Compounds

While the allure of organosulfur-based polymers in biomedical applications shines brightly, a shadow of concern lurks beneath—their potential toxicity. Like a two-faced coin, their promise for therapeutic advancements hinges on navigating this critical safety aspect. Delving deeper requires dissecting the multifaceted nature of this toxicity, with insights from relevant research paving the way for responsible development and application.

### 6.3. Unmasking the Threats

- Monomer Mischief: The very building blocks of these polymers, the sulfur-containing monomers, can pose the first challenge. Some, like bisphenol A (BPA), raise concerns about endocrine disruption and potential carcinogenicity [50]. Others, like thiols, may exhibit cytotoxicity at high concentrations [51]. Choosing safe and biocompatible monomers becomes the first line of defense in minimizing polymer toxicity.
- Degradation Dilemmas: The breakdown of these polymers within the body, while seemingly benign, can unleash hidden dangers. Breakdown products, depending on their structure and reactivity, might exert negative effects on surrounding tissues or cells. Understanding the degradation pathways and ensuring harmless byproducts remain paramount [52].
- Immune Intrigue: The body’s intricate immune system, ever vigilant against invaders, may misinterpret these polymers as foreign threats. Triggering an inflammatory response or even generating antibodies can lead to adverse reactions and hamper the polymer’s therapeutic potential [53]. Tailoring the polymer design to minimize immunogenicity becomes crucial for successful clinical translation.

### 6.4. Strategies for a Safer Path

- Chemical Camouflage: Mimicking natural biomolecules or incorporating stealthy functionalities can help these polymers evade the immune system’s watchful eye. Strategies like glycosylation or polyethylene glycol (PEG) conjugation can enhance biocompatibility and reduce immunogenicity [54].
- Controlled Demolition: Precisely tuning the polymer’s degradation profile offers another avenue for toxicity control. By designing polymers that degrade only under specific conditions or enzymes, scientists can ensure controlled breakdown and minimize the release of harmful byproducts [55].
- Rigorous Testing: Stringent in vitro and in vivo safety assessments are indispensable. Thoroughly evaluating cellular interactions, cytotoxicity, and potential genotoxicity ensures the development of safe and reliable polymers for clinical use [56].

### 6.5. The Road Ahead

While concerns surrounding the toxicity of organosulfur-based polymers are valid, they should not dampen the enthusiasm for their potential. By embracing a safety-first approach, meticulous design strategies, and rigorous testing, scientists can unlock the true therapeutic potential of these versatile materials. Only with continued research and responsible development can this two-faced coin reveal its brighter side, paving the way for safer and more effective treatments in the future.

### 6.6. The Variability in the Properties of Organosulfur-Based Polymers

The versatility of organosulfur-based polymers lies in their diverse structures and compositions, which act like a chameleon’s colorful skin, dictating their ability to encapsulate and release drugs. Delving into this variability unveils a fascinating interplay between polymer design and drug delivery characteristics.

### 6.7. Structural Shapeshifters

The polymer’s backbone architecture dramatically impacts its morphology and interactions. Linear chains often form micelles or vesicles, suitable for encapsulating hydrophobic drugs, while branched structures create gel-like networks ideal for the controlled release of hydrophilic molecules [57]. Combining different sulfur-containing monomers within the same chain opens a Pandora’s box of possibilities, as seen in amphiphilic block copolymers with hydrophilic and hydrophobic blocks facilitating the encapsulation of diverse drugs and tailoring release profiles based on environmental stimuli [58]. Introducing covalent linkages between polymer chains alters their rigidity and degradation behavior, with controlled cross-linking enhancing stability and prolonging drug release, while excessive cross-linking can hinder drug accessibility [59].

### 6.8. Compositional Kaleidoscope

The choice of sulfur-containing monomers plays a crucial role. Thiols, for instance, readily undergo disulfide exchange reactions, enabling controlled release triggered by redox conditions within the body [60]. Sulfonates and sulfates, on the other hand, add water solubility, enhancing biocompatibility and facilitating drug loading for targeted delivery [61]. Decorating the polymer backbone with specific functional groups can fine-tune its interactions with drugs and biological environments. Cationic groups electrostatically bind to negatively charged drugs, while pH-sensitive groups trigger release under specific acidic or basic conditions [62].

This intricate interplay between structure and composition directly impacts drug encapsulation and release. Tailoring the polymer’s size, shape, and functional groups optimizes drug loading capacity and minimizes leakage, ensuring strong interactions and stable encapsulation by precisely matching the chemical properties of the drug and polymer [63]. The polymer’s degradation profile, influenced by cross-linking, functional groups, and environmental factors, governs the rate and pattern of drug release. Slow degradation in biocompatible polymers ensures sustained release, while stimuli-responsive systems offer on-demand delivery triggered by specific signals.

Understanding the impact of structural and compositional variability empowers researchers to design organosulfur-based polymers with tailor-made drug delivery characteristics. This opens doors for personalized medicine, targeting specific drugs to disease sites with controlled release profiles optimized for individual needs. By embracing the chameleon-like nature of these polymers, we can harness their full potential in revolutionizing drug delivery systems.

The characteristics of organosulfur polymers cause them to be attractive candidates for drug delivery structures. Their biodegradability permits a managed and safe drug release, while stimuli-responsiveness enables focused and controlled drug transport. The capacity to encapsulate various medications complements their versatility and applicability. Also, the effect of sulfur-containing functional groups on drug release kinetics means that the release profiles of pills that are enclosed can be customized [64]. Understanding and harnessing these precise residences of organosulfur polymers contributes to improving efficient and targeted drug delivery structures with improved healing results.

## 7. Biocompatibility of Organosulfur Polymers

Ensuring the biocompatibility of drug delivery structures is of the utmost importance in pharmaceutical studies. The protection and compatibility of those systems with biological entities are essential to their successful translation into scientific programs. In this part, we look into the biocompatibility parts of organosulfur-based polymers to show how well they can deliver drugs safely and effectively [65].

First and most importantly, comparing the cytotoxicity of organosulfur polymers is essential to assessing their biocompatibility. Researchers have extensively analyzed the effect of these polymers on cell viability and cellular capabilities. Cell viability assays, including the MTT or the resazurin assay, have been applied to determine the cytotoxic potential of organosulfur polymers. These studies have consistently proven the low cytotoxicity of these polymers, indicating their compatibility with biological systems.

In addition to cytotoxicity, the immunogenicity of organosulfur polymers is another vital element to consider. Drug transport systems can appreciably affect the efficacy and safety of organosulfur polymers by triggering an immune reaction. Researchers have extensively evaluated the immunogenicity of organosulfur polymers. Studies have shown that these polymers showcase minimum immunogenicity, making them appropriate for drug delivery programs [66]. Organosulfur polymers exhibit low immunogenicity due to their biocompatible nature and the absence of immunostimulatory functional groups.

Biodegradability is another crucial factor in biocompatibility when considering drug transport structures. Organisms have developed mechanisms to correctly metabolize and do away with foreign substances, which include polymers. Organosulfur polymers, with their inherent biodegradability, provide a bonus in their compatibility with biological structures. These polymers can undergo degradation into smaller, biocompatible fragments, decreasing the danger of long-term accumulation and potential toxicity [67]. The biodegradability of organosulfur polymers allows for the managed and safe release of medication, ensuring favorable healing outcomes while minimizing unfavorable results.

Furthermore, the ability of organosulfur polymers to impact particular cellular functions ought to be evaluated to determine their overall biocompatibility. For instance, studies have investigated the effect of these polymers on mobile adhesion, proliferation, and differentiation. These tests help us find out if organosulfur polymers change the way cells behave in a bad way and if they can help or hinder specific cell processes that are important for drug delivery.

Safety is an essential consideration for any drug transport device. This part of the research paper looks at how biocompatible and toxic organosulfur polymers are. It talks about how they can change the way cells work and how biocompatible they are in general. Considerable research consistently shows that those polymers have the capacity for secure and robust drug transport, as evaluated for their cytotoxicity, immunogenicity, and biodegradability [68]. Organosulfur polymers are biodegradable, do not hurt cells much, and do not cause immune responses. This shows that they are compatible with living systems and can be used as safe and reliable drug carriers.

By knowing and assessing the biocompatibility aspects of organosulfur polymers, researchers could make knowledgeable decisions and design drug delivery systems that prioritize protection and efficacy. The comprehensive biocompatibility evaluation ensures that organosulfur polymers may be applied confidently in scientific packages, facilitating the improvement of advanced drug delivery systems that provide progressed therapeutic results [69].

Biodegradability is an important property of organosulfur polymers that contributes to their biocompatibility and suitability for drug delivery systems. It is important to know how these polymers break down in order to figure out how they can affect biological structures and create controlled drug release systems.

Organosulfur polymers showcase inherent biodegradability due to the presence of sulfur-containing, purposeful compounds in their shape. These functional groups, which include disulfide bonds or thiol (-SH) groups, are vulnerable to degradation in physiological situations or in the presence of specific enzymes [70]. The biodegradation of organosulfur polymers can happen through different instruments, like enzymatic corruption, hydrolytic cleavage, or oxidative debasement.

Enzymatic corruption is one of the essential pathways for the biodegradation of organosulfur polymers. Some proteins, as well as glutathione, cysteine, or thioredoxin reductase, can break disulfide bonds in the polymer spine [71]. Enzymatic reactions break down the polymer into smaller, more biocompatible pieces that the body can quickly metabolize and eliminate. The enzymatic degradation of organosulfur polymers allows precise manipulation over drug release. Precise enzymes in specific tissues or cell cubicles can trigger the polymer’s degradation and next drug release.

Hydrolytic cleavage is another mechanism of biodegradation for organosulfur polymers. When water breaks down ester or amide bonds in the structure of a polymer, the polymer can be broken down into its individual monomers. The charge of hydrolysis may be inspired by factors that include pH, temperature, and the presence of particular ions or enzymes [72]. By manipulating those factors, researchers can lay out organosulfur polymers with tailor-made degradation fees, allowing for managed and sustained drug releases.

Organosulfur polymers undergo oxidative degradation due to the presence of sulfur atoms. Under oxidative situations, together with those observed in certain cell booths or in the presence of reactive oxygen species (ROS), the sulfur-containing purposeful agencies can go through oxidation. This oxidation can result in the cleavage of the polymer backbone and subsequent degradation into smaller fragments. Organosulfur polymers can break down in oxidative environments, which can be great for drug delivery systems that are focused on specific oxidative environments in the body, like inflamed tissues or tumor microenvironments.

The biodegradability of organosulfur polymers offers numerous blessings in drug delivery. First, the fact that these polymers can break down naturally means that they will be removed from the frame after the drug is released. This reduces the chance of long-term buildup and possible toxicity. The enzymatic, hydrolytic, and oxidative degradation pathways ensure medication’s safe and green release while minimizing potential damaging effects [73]. By knowing how and harnessing the biodegradability of these polymers, researchers can lay out drug transport structures with improved healing effects and decreased toxicity.

## 8. Controlled Release Strategies

The controlled release of drugs is a vital requirement for effective therapy. The capability to precisely manage the release kinetics of drugs allows for the most fulfilling dosing, sustained healing outcomes, and reduced facet results. In this segment, we explore the numerous techniques employed to achieve managed release using organosulfur-based polymers. We delve into the design standards, mechanisms, and elements influencing the release kinetics. Furthermore, we look at the ability packages of these techniques in exclusive healing regions.

### 8.1. Design Principles

The layout of managed release structures using organosulfur polymers entails paying careful attention to numerous vital principles. These ideas guide choosing appropriate polymer systems and component strategies to achieve the preferred release profiles. Some important design standards include the following:

Polymer Selection: The choice of organosulfur polymer is crucial as it determines the overall release behavior. Factors consisting of polymer solubility, degradation charge, and compatibility with the drug of interest must be considered [74].

Drug Loading: The polymer matrix’s method and quantity of drug loading affect the release kinetics. Strategies consisting of physical entrapment, chemical conjugation, or encapsulation within nanoparticles can be employed to achieve managed drug loading.

Polymer Matrix: The polymer matrix’s physical and chemical residences affect medicine’s diffusion and release. Adjusting polymer concentration, molecular weight, and cross-linking density parameters tailors the matrix to modulate the release kinetics [75].

### 8.2. Mechanisms of Controlled Release

Several mechanisms are employed to manage medication releases from organosulfur-based total polymers. These mechanisms determine the release behavior and can be categorized as diffusion-controlled, degradation-controlled, or stimuli-responsive.

Diffusion through the polymer network aids in releasing drugs from the polymer matrix in a diffusion-managed release. The release charge is governed by factors such as drug solubility, polymer morphology, and the awareness gradient [76]. Various polymer architectures, including micelles, hydrogels, or nanoparticles, can also modulate the diffusion-managed release.

Degradation-managed release involves the degradation of the polymer matrix, which is mainly responsible for releasing encapsulated drugs. Organosulfur polymers can be designed to degrade in response to specific stimuli, including enzymatic interest, pH adjustments, or redox reactions. This managed degradation allows for the sustained and precipitated release of drugs [77].

Stimuli-responsive release structures use external triggers, such as temperature, light, or magnetic fields, to achieve a controlled drug release [78]. Incorporating stimuli-responsive elements into the polymer matrix enables the selective activation of drug release at the desired time and location, thereby enhancing therapeutic efficacy.

## 9. Factors Influencing Release Kinetics

Several elements affect the release kinetics of medicine from primarily organosulfur-based polymer systems. These elements want to be taken into consideration in the course of the design and method procedures to obtain the favored release profiles. Some of the important factors encompass those listed below.

The molecular weight, hydrophobicity, and price of the polymer are some of the physicochemical properties that can affect how the drug interacts with the polymer and how quickly the drug is released. The characteristics of the drug, including solubility, molecular weight, and stability, affect its release from the polymer matrix. The composition of the polymer matrix, inclusive of the presence of components, cross-linkers, or co-polymers, can affect the release kinetics by changing the diffusion or degradation properties [79,80]. External factors such as temperature, pH, and enzymatic activity can impact the release kinetics by affecting the polymer matrix or drug–polymer interactions.

## 10. Applications in Therapeutic Areas

Controlled release techniques and primarily organosulfur-based polymers have proven their super capacity in diverse therapeutic areas. The potential to tailor the release profiles of medication permits advanced treatment effects and decreased dosing frequency. Some of the capability packages consist of the following:

### 10.1. Cancer Therapy

Controlled release structures may supply anticancer capsules to tumor sites, maximizing their efficacy while minimizing systemic toxicity. The stimuli-responsive release mechanisms may be especially useful in focusing on tumor microenvironments [81,82].

### 10.2. Cardiovascular Therapy

At a sustained price, organosulfur-based polymers may be used to manage medication release to prevent restenosis or supply cardiovascular therapeutics, along with antiplatelet marketers or vasodilators.

### 10.3. Antimicrobial Therapy

Controlled release structures may be utilized to supply antimicrobial pills for localized infections or to provide an improvement of antibiotic resistance by maintaining healing drug tiers over an extended period [83,84].

### 10.4. Wound Healing

The controlled release of boom or wound healing factors from organosulfur polymers can promote tissue regeneration and boost wound recuperation procedures.

## 11. Nanotechnology in Organosulfur Polymers

Nanotechnology has revolutionized the drug delivery field by enabling the precise manipulation of drug release and concentration. Integrating nanotechnology with primarily organosulfur-based polymers allows for development of advanced drug transport systems with more robust therapeutic efficacy and the advancement of patient results. In this phase, we explore nanoscale drug transport systems’ fabrication strategies and applications, emphasizing the sizable improvements accomplished by combining nanotechnology and organosulfur polymers [85,86].

Fabrication techniques using organosulfur polymers to make nanoscale drug transport systems requires a lot of different techniques that let you control the particle length, shape, and drug loading ability in unique ways. Some of the generally employed fabrication techniques consist of the following:

Nanoprecipitation is a versatile approach to fabricating nanoparticles by rapidly blending a polymer with an antisolvent. This system ends in the formation of polymer nanoparticles with high drug loading capacity and controlled release properties [87]. In this approach, the evaporation of the immiscible solvent is used to observe the emulsification of a polymer solution containing a drug, resulting in drug-loaded polymer nanoparticles with a controlled size distribution. This process results in drug-loaded polymer nanoparticles with a controlled size distribution [88].

Electrospinning is a technique used to manufacture nanofibers from polymers. Using excessive voltage in a polymer solution, an excellent polymer jet is electrostatically drawn and accumulated as nanofibers, which can be similarly functionalized for drug transport packages [89,90].

Self-assembly techniques contain the spontaneous employment of polymers into nanostructures, such as micelles or vesicles, in the presence of drugs or different molecules. This method allows the formation of well-defined nanostructures with controlled drug-release properties.

## 12. Applications of Nanoscale Drug Delivery Systems

Integrating nanotechnology with primarily organosulfur-based polymers has led to significant improvements in drug delivery structures, presenting advanced healing effects in diverse packages. Some of the essential programs include the following:

### 12.1. Targeted Drug Delivery

Nanoscale drug transport structures allow for the targeted delivery of drugs to unique sites in the frame. Functionalizing the nanoparticles’ surface with targeting ligands, such as antibodies or peptides, enables the selective delivery of drugs to specific cells or tissues, thereby improving healing efficacy and minimizing off-target effects [91]. Poly(ε-caprolactone)-β-poly(ethylene glycol)-β-poly(ε-caprolactone) triblock copolymers were functionalized with β-cyclodextrin for the targeted delivery of doxorubicin to cancer cells. The cyclodextrin–polymer complex exhibited enhanced cellular uptake and cytotoxicity in vitro compared to free doxorubicin [92].

### 12.2. Better Drug Stability

Putting medicine inside organosulfur-based polymers at the nanoscale level protects it from breaking down and makes the drug more stable, especially for pills that do not dissolve easily or are easily broken down by enzymes. This improved stability guarantees the most reliable drug delivery and bioavailability [93].

Incorporating nanotechnology into organosulfur polymers allows particular control over the release kinetics of medication. A researcher can change the release rate and obtain sustained drug release over a long period of time by changing the nanoparticles’ length, composition, or surface charge. Functionalizing these nanoparticles with imaging probes or contrast markers enables the non-invasive imaging of unique disease sites and facilitates early prognosis [94].

The integration of nanotechnology with organosulfur-based total polymers has tremendous potential in the subject of drug transport. The fabrication techniques and packages discussed in this segment show the versatility and blessings of nanoscale drug delivery systems. By combining the precise properties of organosulfur polymers with nanotechnology, researchers can expand enormously efficient drug delivery systems that improve therapeutic efficacy, enhance patient compliance, and limit aspect effects [95,96].

## 13. Future Prospects

Organosulfur-based polymers have shown tremendous ability as drug delivery systems, but there are many exciting possibilities for exploration and innovation. The future possibilities of those polymers in drug delivery include the following: 

### 13.1. Enhanced Targeting Strategies

Combining organosulfur-based total polymers with new focused methods, such as ligand–receptor interactions or structures that respond to stimuli, shows great potential for delivering drugs precisely and effectively to specific types of cells or tissues. Further research in this area can lead to the development of mainly targeted therapies with minimum off-target effects [97].

### 13.2. Personalized Medicine Approaches

These aim to tailor clinical remedies to sufferers primarily based on their precise characteristics, consisting of their genetic makeup or ailment country. Primarily organosulfur-based polymers can be applied in customized medication techniques by incorporating patient-specific factors into the layout and components of drug delivery structures. This personalized approach can optimize remedy outcomes and reduce damaging effects [98].

### 13.3. Combination Therapy

This has shown notable promise in treating complicated diseases, where a couple of capsules or therapeutic agents are taken simultaneously. Primarily organosulfur-based polymers can facilitate co-transporting a couple of drugs with exclusive release kinetics, enabling synergistic results and improved therapeutic effects. Further exploration into mixture healing procedures using these polymers can cause breakthroughs in sickness treatment.

## 14. Challenges and Considerations

To facilitate the scientific translation of organosulfur-based polymers, it is necessary to address numerous challenges and concerns. Some of the essential challenges and concerns encompass the following:

The biocompatibility and safety of primarily organosulfur-based polymers are of the most importance for clinical treatments. Further research is necessary to comprehensively compare their long-term biocompatibility, immunogenicity, and toxicity to specific cell types and organs [99].

The scalability and manufacturing of primarily organosulfur-based polymer synthesis and production techniques want to be optimized. Improving cost-powerful and scalable production methods is vital for translating these polymers into commercial drug transport systems.

Regulatory approval for medical translation is a massive task. Extensive preclinical and scientific studies are vital to establish the protection and efficacy of primarily organosulfur-based polymer drug delivery systems, which may be time-consuming and resource-extensive [100].

The stability and shelf life of organosulfur-based polymer formulations must be carefully evaluated to ensure the encapsulated pills’ long-term efficacy and storage stability. Factors including degradation, drug release kinetics, and storage conditions must be considered.

## 15. Identifying Research Gaps

### 15.1. Biodegradability and Clearance Mechanisms

While primarily organosulfur-based polymers have proven to be promising in drug transport systems, we may want to look further at their biodegradability and clearance mechanisms in vivo. The development of more secure and extra-biocompatible drug delivery systems can be achieved by understanding the degradation and clearance mechanisms of these polymers [101].

### 15.2. Long-Term Stability and Drug Release Kinetics

More research needs to be carried out on the long-term stability of organosulfur-based total polymer formulations and how quickly they release drugs. Determining the stability of these polymers over prolonged intervals and understanding the factors that impact drug release kinetics can be useful in the design of drug delivery structures with the best healing consequences.

### 15.3. Targeting Strategies and Ligand–Receptor Interaction

Using organosulfur-based total polymers has proven to have promising outcomes with a focus on targeting strategies. There is a need to explore and optimize the layout of ligands for unique targeting. Targeting techniques can be more specific and effective if we study how ligands and receptors interact and how that affects how well drugs move through cells.

### 15.4. In Vivo Efficacy and Therapeutic Outcomes

Although in vitro studies have confirmed the ability of organosulfur-based polymers in drug delivery, there may be a need for extra comprehensive in vivo research to evaluate their efficacy and therapeutic outcomes. Conducting animal studies and ultimately clinical trials can offer valuable insights into the safety and efficacy of these drug delivery structures in real-world settings [102].

### 15.5. Scalability and Manufacturing Processes

The fields progresses to medical translation, there is a desire to explore scalable and reproducible manufacturing tactics for organosulfur-based polymers. Optimizing manufacturing techniques can ensure consistency within those polymers’ properties and overall performance, facilitating their business manufacturing and considerable use.

### 15.6. Combination Therapies and Synergistic Effects

The capacity for mixed treatments using organosulfur-based polymers is a place that calls for additional research. Exploring the synergistic effects of various drugs or healing dealers while bringing the use of these polymers can lead to the improvement of extra powerful treatment strategies for complicated sicknesses.

Addressing those research gaps will contribute to developing organosulfur-based polymers in drug transport systems. Researchers can triumph over current limitations and increase extra green and targeted drug delivery systems by exploring biodegradability balance and concentrating on strategies, in vivo efficacy, manufacturing methods, and combination healing procedures. Those gaps can be addressed through interdisciplinary collaborations and persevering research efforts, paving the way for future breakthroughs in organosulfur-based polymer drug delivery structures [103].

## 16. Conclusions

The present review provides a comprehensive insight into the current state of research on organosulfur-based polymers as drug delivery vehicles. The special qualities of these polymers make them very useful for controlled drug delivery, release, and targeted treatments. However, further research is necessary to enhance the performance of these systems in challenging situations and in medical applications. Studies have shown that organosulfur-based polymers hold significant potential as drug transport structures. Moreover, this study describes an in-depth look in the latest progress in many integrated areas, such as synthesis, characterization, and biocompatibility, controlled release, nanotechnology, and targeted therapy. By identifying research gaps, we hope to encourage further research work that will help in better understanding organosulfur-based polymers and innovative approaches towards their applications as drug delivery components.
